# A focus groups study of staff team experiences of providing interdisciplinary rehabilitation for people with dementia and their caregivers—a co-creative journey

**DOI:** 10.1186/s12877-023-04269-3

**Published:** 2023-09-18

**Authors:** Nina Lindelöf, Ingeborg Nilsson, Håkan Littbrand, Yngve Gustafson, Birgitta Olofsson, Anncristine Fjellman-Wiklund

**Affiliations:** 1 Department of Community Medicine and Rehabilitation, Physiotherapy, Umeå University, Umeå, SE-90187 Sweden; 2 Department of Community Medicine and Rehabilitation, Occupational therapy, Umeå University, Umeå, Sweden; 3 School of Health and Welfare, Halmstad University, Halmstad, Sweden; 4 Department of Community Medicine and Rehabilitation, Geriatric Medicine, Umeå University, Umeå, Sweden; 5 Department of Nursing, Umeå University, Umeå, Sweden; 6 Department of Surgical and Perioperative Sciences, Umeå University, Umeå, Sweden

**Keywords:** Dementia, Rehabilitation, Person-centered care, Informal caregiver, Interdisciplinary health team, Experiences, Grounded theory

## Abstract

**Background:**

The World Health Organization claims that rehabilitation is important to meet the needs of persons with dementia. Rehabilitation programmes, however, are not routinely available. Person-centred, multidimensional, and interdisciplinary rehabilitation can increase the opportunities for older adults with dementia and their informal primary caregivers to continue to live an active life and participate in society. To our knowledge, staff team experiences of such rehabilitation programmes, involving older adults with dementia and their informal caregivers has not been previously explored.

**Methods:**

The aim of this qualitative focus group study was to explore the experiences of a comprehensive staff team providing person-centred multidimensional, interdisciplinary rehabilitation to community-dwelling older adults with dementia, including education and support for informal primary caregivers. The 13 staff team members comprised 10 professions who, during a 16-week intervention period, provided individualised interventions while involving the rehabilitation participants. After the rehabilitation period the staff team members were divided in two focus groups who met on three occasions each (in total six focus groups) and discussed their experiences. The Grounded Theory method was used for data collection and analysis.

**Results:**

The analysis resulted in four categories: *Achieving involvement in rehabilitation is challenging, Considering various realities by acting as a link, Offering time and continuity create added value*, and *Creating a holistic view through knowledge exchange*, and the core category: *Refining a co-creative process towards making a difference.* The core category resembles the collaboration that the staff had within their teams, which included participants with dementia and caregivers, and with the goal that the intervention should make a difference for the participants. This was conducted with flexibility in a collaborative and creative process.

**Conclusions:**

The staff team perceived that by working in comprehensive teams they could provide individualised rehabilitation in creative collaboration with the participants through interaction, knowledge exchange, time and continuity, coordination and flexibility, and a holistic view. Challenges to overcome were the involvement of the person with dementia in goal setting and the mediating role of the staff team members. The staff pointed out that by refinement they could achieve well-functioning, competence-enhancing and timesaving teamwork.

**Supplementary Information:**

The online version contains supplementary material available at 10.1186/s12877-023-04269-3.

## Background

Rehabilitation in comprehensive interdisciplinary teams is recommended when an individual’s problems are complex [1] and has shown promising effects [2–4]. Person-centred multidimensional interdisciplinary rehabilitation refers to the process whereby a team, consisting of many different professions, conduct comprehensive assessments to identify problems and needs, strengths and resources of an individual. In interdisciplinary team rehabilitation the goals are set together with the patient and, when such is the case, the informal primary caregiver [1, 5]. Based on the goals, interventions are implemented, and continuous follow-ups are performed [2]. There is a close overlap between interdisciplinary rehabilitation and a person-centred approach [2]. The latter implies that every individual is unique; the person is the focus, not the disease e.g., in the case of a dementia diagnosis [6]. Since a person has different wants, feelings, resources, and needs, it is important to adapt interventions individually and to engage the person as an active participant in their own rehabilitation [6, 7].

Adults with dementia (i.e., neurocognitive disorders) are a heterogeneous group, with a variety of complex problems and needs caused by this progressive and long-lasting condition. The complexity includes limited awareness of difficulties in everyday life and anxiety regarding upcoming events [8], which can pose challenges during the implementation of a rehabilitation programme. Although many adults with dementia live in nursing homes due to dependency in personal activities in daily living (ADL), ordinary housing is the most common way of living with dementia in Sweden [9]. In managing everyday life in ordinary housing, a heavy burden is put on informal caregivers [10]. These circumstances must be given special consideration in rehabilitation programmes for older adults with dementia who are living in ordinary housing. It is a challenge to meet the complex needs and, thus, there is a need that the rehabilitation is conducted by a comprehensive and multi-professional team, with a person-centred and interdisciplinary approach. However, interventions with this approach among people living in ordinary housing and having dementia are sparsely studied [2, 11, 12].

Interdisciplinary rehabilitation has the potential to increase the opportunity for older adults with dementia and their caregivers to continue to live an active life and participate in society. The World Health Organization (WHO) claims that rehabilitation is important to meet the needs of the affected persons [13]. However, in contrast to rehabilitation for people with other diseases engaging the central nervous system, rehabilitation programmes are not routinely available for older adults with dementia [14–16]. According to WHO the gap between the need for rehabilitation and its implementation depends on lack of awareness and understanding of dementia, resulting in stigmatization and barriers to care [13]. In interviews, health professionals perceived that barriers exist regarding the ability of patients with dementia to participate in rehabilitation. They expressed difficulties in defining worthwhile outcomes and believed that achievable outcomes were not sufficiently worthwhile to invest in [14].

Based on knowledge of the complexity of dementia disorders in our research group and positive findings in previous rehabilitation studies among older adults with dementia [3, 4, 17, 18], we expanded the present rehabilitation programme to include teams with many different professions. The rehabilitation programme has been evaluated for experiences among participants with dementia [19]. Results show that self-esteem, motivation and self-efficacy seemed strengthened by daring and coping, collaborating in the group, and being seen by the staff. Insights into dementia raised concerns about the future, but also served as incentives to continue with prioritized activities [19]. Additionally, it is important to explore staff team experiences of this type of intervention to increase knowledge about the feasibility and implementation of interdisciplinary rehabilitation programmes for adults with dementia and their caregivers. The staff team’s experiences would also provide important knowledge for future planning and development of rehabilitation programmes for this population. To our knowledge, the experiences of working in a comprehensive team focusing on rehabilitation involving older adults with dementia and their informal caregivers have not been previously studied.

The aim of this study was to explore the staff team experiences of providing person-centred multidimensional, interdisciplinary rehabilitation to community-dwelling older adults with dementia, including education and support to informal primary caregivers.

## Methods

To address the aim of this study, we used a qualitative research design with focus groups [20–22], analyzed using the Grounded Theory (GT) method [23, 24]. GT is considered suitable when a new intervention is evaluated, including processes over time and complex factors influencing health and illness [25]. We consider this is the case in the present study, where an extended rehabilitation team, in the context of a larger trial, is delivering a new intervention for older adults with dementia and their informal caregivers, i.e., family or relatives and others, such as neighbours or friends who support the participant with dementia.

### Context of the study and the staff team’s shared experiences

The focus groups included staff who had shared experiences from the Multidimensional InterDisciplinary REhabilitaion in Dementia study (the MIDRED study), targeting community-dwelling older adults with dementia and their informal primary caregivers. The MIDRED study is a randomized controlled pilot study conducted in Umeå that is a middle-sized university town in northern Sweden. In the study a person-centred, multidimensional, and interdisciplinary rehabilitation programme is being evaluated both quantitatively and qualitatively. The programme has been developed within the trial for community-dwelling older adults with dementia and includes education and counseling of informal primary caregivers. In the intervention group there were 31 participants with dementia (PwDs) and 35 informal primary caregivers. To date, there is one qualitative study published based on the MIDRED study [19]. The study protocol is registered at Current Controlled Trials Ltd, ISRCTN59155421. Date of registration 04/11/2015.

**Fig. 1:**
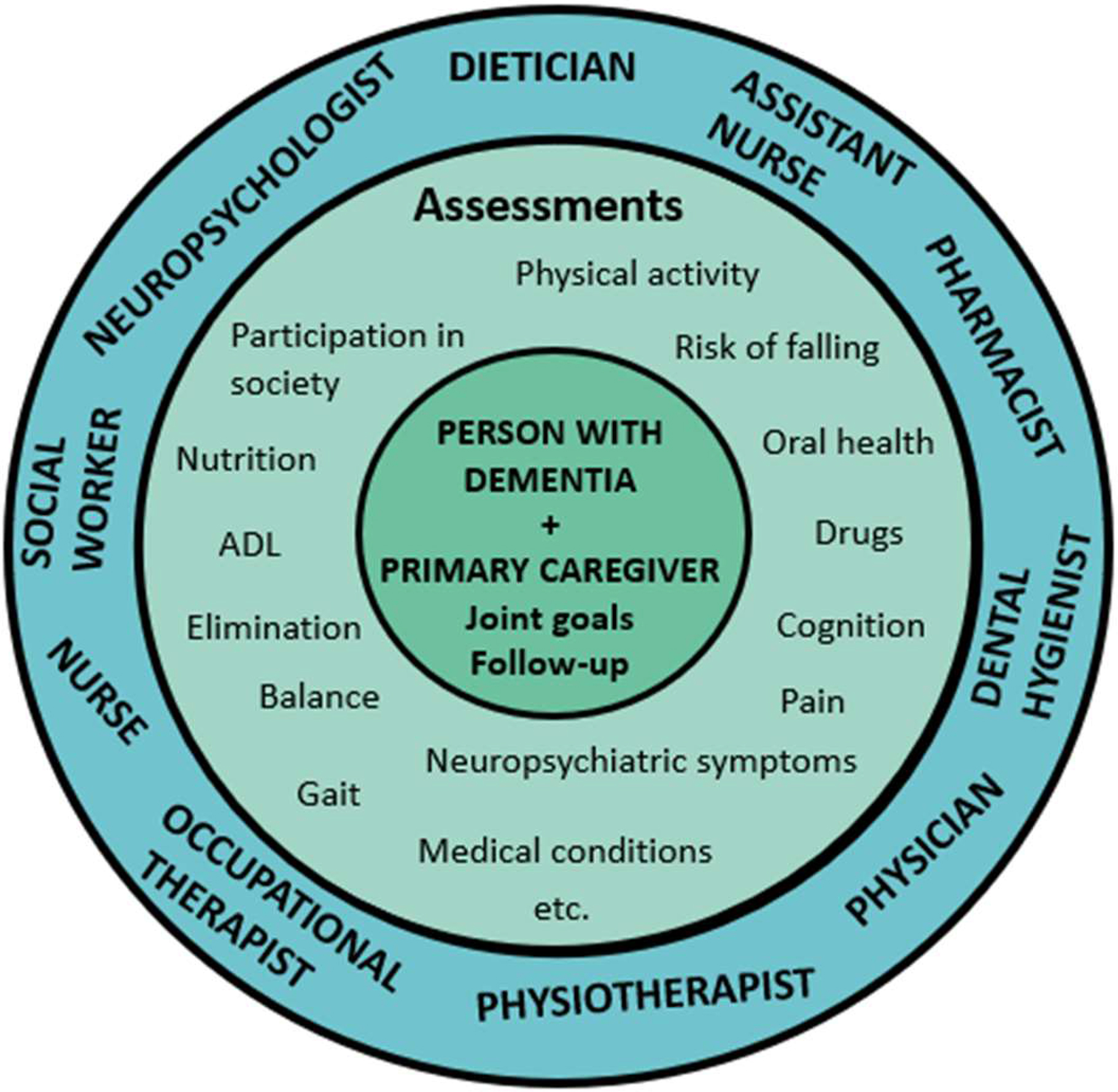
The comprehensive team, including the participant with dementia and the informal primary caregiver, as well as examples of assessments and potential areas for intervention

An intervention period of 16 weeks was preceded by four weeks of assessments where professions in the comprehensive team identified both resources and needs of each PwD in potential areas for intervention (Fig. 1). The informal caregivers’ need for individual support and counselling was also identified. Based on the findings, representatives of the staff team, together with each PwD and her/his informal primary caregiver(s), set up a rehabilitation plan with individual rehabilitation goals. They also planned specific interventions, and relevant professions formed a smaller team for each PwD.

Continuous follow-ups were conducted, and the staff team also met once a week for team conferences where they evaluated goal completion, how the interventions were progressing, and whether new problems had arisen for the PwDs or their caregivers. Five and 14 months after the rehabilitation period, respectively, they were reassessed by the staff team according to the same routine as at baseline. Earlier recommended interventions were followed up and complementary interventions could be initiated.

Examples of individual-based interventions led by relevant professionals were medical controls, prescription of cognitive technical devices, introducing a PwD to a day-care centre and other activities in the community, providing support to engage in household activities, and reviewing, advising, and making corrections to medication regimes. Further the professional team provided support to prevent malnutrition, interventions related to oral care, psychological support, social support regarding home services and economy, and support to informal primary caregivers for coping with the consequences of dementia in daily life. Twice a week each PwD was offered individualised exercise based on The High-Intensity Functional Exercise (HIFE) Programme, with the goal of improving lower limb muscle strength, balance, and mobility [26, 27]. The exercise sessions were conducted in small groups and afterwards the groups met for a coffee break with social interaction. PwDs and caregivers also received individual recommendations and guidance for achieving physical activity levels in accordance with global health promotion recommendations [28]. The informal primary caregivers were offered six group sessions consisting of education and discussions about dementia, and individual support from the social worker when needed. The interventions were conducted in rehabilitation facilities at the geriatric clinic, in the homes of PwDs with dementia, and/or in the community. The staff team organized transportation to the clinic, assisted PwDs when they arrived at and left the clinic, and participated in coffee breaks after the exercise sessions to ensure that participants felt confident and welcomed.

The staff worked in different constellations to individualise the rehabilitation for each participant, based on their goals and rehabilitation plans. The staff team had close collaboration within the team, including collaboration with the PwD and their informal caregivers, as well as with other relatives. They also collaborated with other actors, such as different health care providers, the municipality, dental clinics, day-care centres, exercise groups in the community, and home social services.

### Participants

The participants in the present GT study were all staff team members of the MIDRED study, including the professions: assistant nurse, dental hygienist, dietician, neuropsychologist, nurse, occupational therapist, pharmacist, physician, physiotherapist, and social worker. In total 13 staff team members were invited, and all accepted to participate and share their experiences from the interdisciplinary teamwork. At the time of the focus groups the participants had been part of the planning and implementation of the interventions. They are not researchers in the present study.

The focus group participants were divided into groups (A and B groups) with six and seven participants, respectively. No group included more than one participant from a profession. The professional team consisted of twelve women and one man with a median age of 47 (35–65) years. They had a median of 19 (2.5–40) years of experience in their profession and 5 (1.5–35) years of experience working with adults with dementia.

### Data collection

Six focus groups, three sessions with each group, were conducted between September 2016 and January 2017; the first directly after the end of the intervention period and the second within one month thereafter. The time of the third focus group sessions were after the five-month follow-up conducted by the staff team.

Each focus group started with an introduction to the study, the expectations of the participants (i.e., to describe, reflect and discuss their experiences of the teamwork) as well as a reminder of the ethical principles e.g., confidentiality. Consent forms and demographic information about the participants were collected. In collaboration with all authors, an interview guide for the first and the last focus group was constructed to support the interviews. For details of the interview guide for the first focus group session, see Supplementary file 1. The second interview was constructed to follow up on themes and issues that each group had raised in the first interview. A starting question was mailed to each participant before the second interview started; the focus of this question was based on the first interview. The starting questions in each group were: “*Can you give an example of something that you developed during the course of your work?”* and *“Can you tell us about a situation where you feel the team made a difference?“*, respectively. The third focus group session comprised reflections on, and examples of, what had happened during the interventions and follow-ups as well as a question about their views on the structure of the rehabilitation program.

The focus groups were led by two authors (AFW and IN) with extensive experience of research interviews, clinical rehabilitation, and teamwork. Of the two focus group leaders, one took a more active role (IN), while the other (AFW) was more of an observer who raised follow-up questions and summarized topics that were discussed at the end of each focus group session [22]. They were not involved in the intervention. Each focus group session lasted one and a half hours. We consider that the data is very rich in content from the composition of the focus groups and that saturation was achieved.

All focus groups were audio recorded and transcribed verbatim by a professional transcriber who was not involved in the study. The focus group leaders recorded field notes during and immediately after each focus group session, including reflections on how the focus group went, to get a deeper understanding of important topics and themes. After the first focus group session with each group, field notes and the audiotaped focus group discussions were reviewed to develop a relevant and important interview guide for the second focus group sessions. The first author (NL) was actively involved in preparing and following up each focus group, and the rest of the author team served as discussion partners during the data collection.

### Data analysis

The focus groups were analyzed using the GT method, focusing on constant comparisons [23, 24]. In the first analysis step, three of the researchers (AFW, IN, NL) listened to the recordings and read the transcripts carefully and repeatedly. They performed separate open coding of the transcripts, meaning that the transcripts were read paragraph by paragraph and line-by-line and important information raised was assigned codes on a low abstraction level. Parallel coding was performed for two transcripts (by NL and AFW, and by NL and IN). The open codes were discussed and compared between these researchers for a negotiated outcome. Codes with similar content were compiled into sub-categories and categories on a more abstract interpretation level. Ideas relating to the emerging result were continuously written down in memos, which were used in the data analysis. During the analysis process, a core category was constructed together with the categories described above. In the final analysis step, the categories were compared to identify how they related to each other and were linked in a process. In doing so, the categories were integrated with the core category to conceptualize the process as a theoretical model. The model illustrates the participants’ experiences of providing person-centred multidimensional, interdisciplinary rehabilitation. The model is further integrated with existing concepts and theories in the discussion.

Throughout the whole analysis process, triangulation between researchers was used [23], meaning that the research group analyzed data individually and within the group. The results were from time to time presented and discussed among all authors of the study. A writing-rewriting process, including discussions and reflection among the research group members, refined the analysis. The research group represents various rehabilitation professions and research fields within health science and medicine, geriatrics, nursing, occupational therapy, and physiotherapy. The three researchers who completed the main analysis had a professional background in rehabilitation, and all had experience of qualitative research. The Consolidated Criteria for Reporting Qualitative Research (COREQ) checklist [29] was used as a support to ensure comprehensive reporting and transparency of the study.

## Findings

**Fig. 2:**
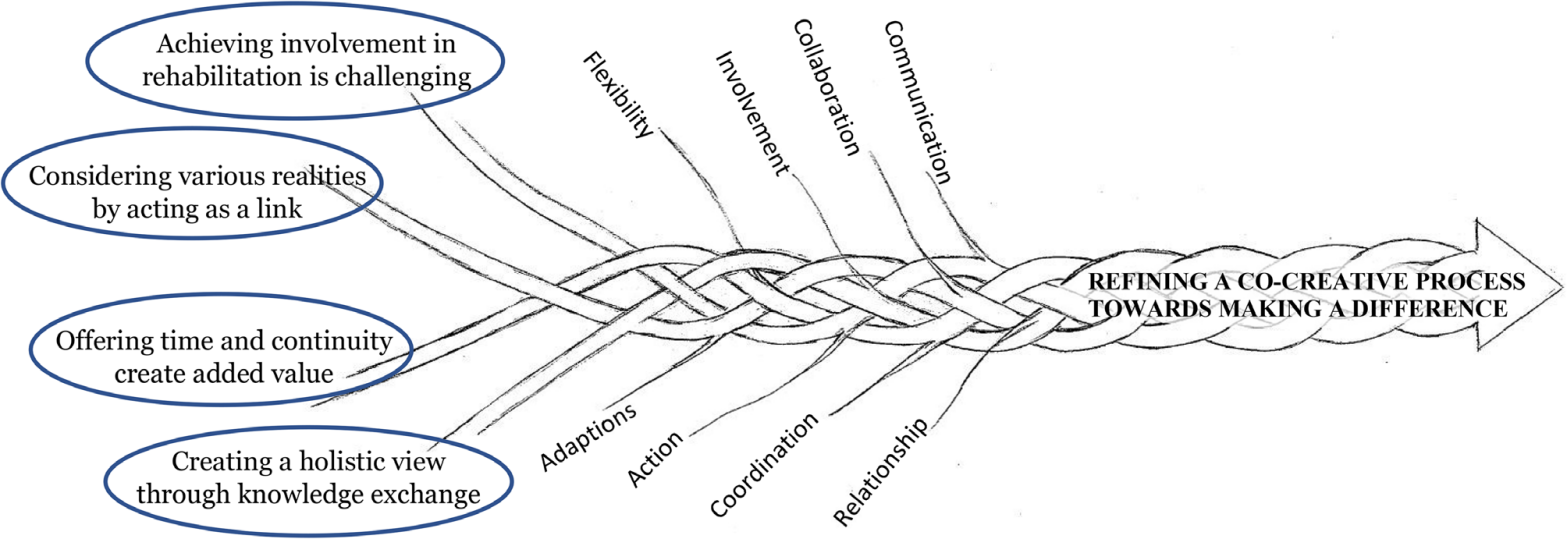
Model of providing person-centred multidimensional, interdisciplinary rehabilitation to community-dwelling older people with dementia and their caregivers showing the context, the categories, and important features leading to the core category

The analysis resulted in one core category ***Refining a co-creative process towards making a difference*** (Fig. 2), and four categories: *Achieving involvement in rehabilitation is challenging, Considering various realities by acting as a link, Offering time and continuity create added value*, and *Creating a holistic view through knowledge exchange.* The categories are presented below, illustrated with quotes from the focus groups. Indicated in brackets after each quote are the two focus groups (A and B) and the interview sessions 1–3. In the first paragraph of each category the content is summarized, and key phrases are written in Italics. The participants are presented anonymously by not mentioning their professions. Furthermore, they are referred to as the staff team to distinguish them from participants in the rehabilitation programme, i.e., PwD and their caregivers.

The categories with their meaning are intertwined and lead to the core category ***Refining a co-creative process towards making a difference***, expressed as a model (Fig. 2). The joint work considers the inter-professional contributions from, and collaboration between, all involved, i.e., the team of staff, the participants with dementia, and the informal primary caregivers. A flexible approach permeates the categories, portraying how the teams navigated and wove their way forward, including both an open mind and pragmatic view to work with a flexible approach. Having an open mind implies that the team of staff identified opportunities rather than obstacles. In that spirit, they adapted themselves and their input when planning and launching actions for the participants in the rehabilitation programme. The way that the team staff collaborated was also a creative and ongoing developmental process, which was refined throughout the rehabilitation period. They focused on working towards common goals, making a difference in the lives of the PwD and their primary caregivers.

*“X: You work together and share knowledge with each other. You get knowledge and you give knowledge and help each other … the patient reaches further thanks to it. So, my goal is the goal that the patient has. I develop by the knowledge exchange and community, so it is much more fun to work that way. So, it gives more energy. That is what I think*.

*Y: I also think that it has been very rewarding to have such competent co-workers from different professions who work together and it is geriatrics in a nutshell to work in teams”.(1B)*.

### Achieving involvement in rehabilitation is challenging

Involvement of the PwDs and their informal caregivers in the rehabilitation was an essential ambition of the rehabilitation programme. However, the focus group participants experienced challenges for this, which are presented in this category. When formulating common rehabilitation goals, the challenges were *to handle impaired insight and low self-esteem* among the PwDs or their caregivers, and *not to offend* by speaking over their heads. Furthermore, engagement could be affected by the relationships between PwDs and their caregivers, where the staff team found *relationships being for better or for worse.* The staff team tackled these challenges through consensus, strategies, and timing.

The question of involvement was most evident when the staff team discussed the importance of setting common goals and suggesting actions. The PwD and the caregiver had to be involved to facilitate both goal setting and the rehabilitation process. However, it was perceived as difficult to get the PwD involved to a degree where they also formulated their own goals based on needs and wishes. Some specific goals were easier to formulate and work towards together with a PwD, such as concrete goals about eliminating different risks, e.g., fall risk, than goals related to increasing quality of life, such as identifying meaningful everyday activities to engage in. In addition to impaired memory, one particular reason for difficulties in goal setting was if a PwD had impaired insight into her/his problems and capabilities. Additionally, it was discussed that low self-esteem impeded the ability to have self-wishes and to formulate goals. The staff team also raised that some PwDs and caregivers did not have such great claims and did not want to bother others and therefore they did not speak up for themselves. The staff team perceived a conflict between setting goals and these problems since they tried to avoid offending by not involving participants or by setting goals over their heads.

*“To work in an interdisciplinary way really requires that it is the patient’s goal. The patient must have formulated the goals, and these must be such things that we all feel that we can work with. So, we support the patient to formulate their goals, but it should be based on their wishes and prioritised activities… and this is difficult to reach, I think, among people with dementia. I think it has been a great challenge.” (2 A)*.

It was also difficult to formulate goals that would satisfy all involved. Both the PwD and the informal caregiver had to be *‘on track’.* The staff team often had to step back and adapt the goals when a participant did not notice the same problems as the staff team had presented. Consensus was seen as a prerequisite to set and formulate goals for the rehabilitation period and involvement from the PwD was perceived as giving the staff team *‘a mandate for action’.*

The staff team reasoned that another option could have been to formulate the main goals later in the rehabilitation period, when the participants and the team of staff members had become familiar with each other. When the participants felt comfortable and safe it was easier for them to make plans and express their wishes and needs. In addition, when participants’ self-confidence had increased, they dared to think that they were able to participate in an activity. At a later stage of the rehabilitation process, participants had the courage to formulate goals for the future. The staff saw it as important to set the goals at the right time to achieve the participants’ best involvement.

*“X: We had an evaluation in the middle somewhere, where we maybe could have been even clearer with capturing new problems, perhaps formulate some new goals. We might have dared to be more specific then when they felt more at home. Actually.*

*I: Adjust the goals as they get a little braver and more…*.

*X: Exactly. To keep that in mind, actually.*

*Y: Yes exactly… if you do not think you can do anything, you rarely dare to dream…*.

*X: But if you have gained the confidence to take a few steps, it may be easier to believe that you can take a few more steps.” (1 A)*.

Sometimes the staff perceived an informal caregiver as over-protective, which resulted in passivity for the PwD. In addition, informal caregivers could have diseases or physical, cognitive, or psychological problems. The caregiver could be speaking for, or instead of the person with dementia, making it difficult for the team to reach the PwD and ensure her/his will and need. The relationships in the family could be for better or worse, and the latter could be difficult for the staff team members to handle.

*“X: Might have a next of kin who supports them or might not. So, there are still… ladies who would feel good by having home care services, for example, or being allowed to leave home for their own activities, but one of them promptly says no and thinks that she is happy just by staying at home and doing nothing. The other has a husband who speaks constantly for her, so it is impossible to work there.*

*Y: And she expressed again that she would like to go to the opera, but he just talks this down.” (3 A)*.

### Considering various realities by acting as a link

In the focus groups, it was expressed that there were many considerations and situations that the staff team members had to deal with. They had to *balance between different expectations* and at the same time the *collaboration gave an extra impact* on the teamwork. During the rehabilitation the staff team were *supplying information, support, and social contact* between different actors and they emphasized that *the team was the link.*

The staff team members sometimes had to act as mediators to manage different wills or “realities” that the PwDs and the caregivers expressed. The staff could sometimes get contradictory information, for example from two siblings. This required a lot of flexibility and creativity from the staff team members. Different opinions could be presented about what was seen as a problem and about what was supposed to be done, e.g., if the greatest need was a new aid in their home or to get out and meet other people. When an informal caregiver was asked, it could sometimes be difficult to distinguish between the need of the PwD and caregiver’s needs. However, the focus groups discussed that much of the well-being of the PwD depended on the fact that the informal caregivers have a crucial role in the life of the PwD. It was emphasized that they do help and support an incredible amount. The intentional and close collaboration with the PwD and their informal caregivers was perceived as having an extra impact on the teamwork.

*“It becomes a kind of synergy effect of the next of kin feeling that they receive support at the same time as the patient [PwD] receives it. Both are in a process forward, which means that you reach a higher level, I think.” (1 A)*.

The staff reasoned that it was not self-evident that a next of kin was the only support the PwD needed. Therefore, it was necessary that these individuals received help from other actors and as expressed in one focus group, ‘*Their legitimate support from society’.* These actors could be the home service, a day-care unit, primary care, or responsible professionals in the community. Communication, flexibility and being a link between these other actors and the PwD together with a caregiver were discussed as necessary since many did not know who to contact and how. It was seen as essential that information was conveyed but the staff team members could not fully rely on the information given when a PwD lived alone and had limited ability to remember or describe problems and needs. It was common that those living alone did not have support when needed. Additionally, there were limitations discovered in the communication and professional attitude and approach of the home service staff, and it was perceived that they had limited knowledge about dementia and therefore, some of the staff team had to act as tutors.

*“There are a lot of treatment problems, I think, among home care staff. They do not know how to deal with dementia in order to carry out the activity as it is intended… And that is a skills gap, I think, that exists in the home care service. So I have had to be the link between the daughter and the home care service to try to get each and everyone’s wishes met.” (3 A)*.

Another example when the staff team members and the interventions served as a link was the group sessions for the informal caregivers. The focus groups reasoned that this social contact space could have a long-lasting impact, and some began to meet outside the group sessions. Others exchanged phone numbers since they wanted to talk about the unusual dementia diagnosis that their partner had, for example. Lastly, in addition to the staff team, the rehabilitation sessions were links between the PwDs when they met in groups twice a week.

*“That the group as such,…that they have become so accepting. ‘She who seems so incredibly wise, she also has such a catastrophic memory’… Being able to share the misery can make it easier.” (3B)*.

### Offering time and continuity create added value

In this category the participants’ experiences of the benefits of having enough time during rehabilitation is described. They meant that *time is a prerequisite for using rehabilitation strategies and for achieving continuity.* Furthermore, it was emphasized that *building relationships takes time*, that enough time was needed *to initiate processes and begin changes* as well as *to provide the conditions for maintenance* of these changes.

The amount of time over a long period, which was the case in this trial and something the staff team members were not used to in their clinical work gave them opportunities to collaborate, change their approach, or make a change towards a more appropriate strategy. This was considered to bring benefits since they had enough time to formulate and revise common goals, try different activities, coordinate actions, and there were opportunities for the professional and the PwD to get to know each other. Enough time came up as a prerequisite for using and refining rehabilitation strategies, such as building relationships, achieving involvement from participants, and initiating processes and beginning change. There was a consensus that a certain amount of time was necessary to create understanding and trust, and for providing the conditions for sustainability.

*“I assume that good work takes time. That is, if it is supposed to be qualitative and it is to be sustainable, then it takes time.” (3B)*.

Enough time gave opportunities to build rehabilitation on the healthy perspectives, strengths, and interests of each participant, to sort out and process problems, and to coordinate and adapt interventions. The PwDs had difficulties to describe their complex problems because of impaired insight and memory and the team of staff experienced it as difficult, to get a picture of the situation after one or two meetings. They reasoned that it was impossible to do what was expected if you only meet a PwD once.

*“X: And it probably takes a lot of commitment to get a good picture and that’s what you have got when you have met them so often here. That is, to add another small piece of the puzzle each time and towards the end you know… you think you know approximately how they are at home and what they need, but the work to get there is quite long. So that work is difficult to do when you meet them once, as you normally do. It is important that it is a team if one should have the opportunity to get it reasonably right.*

*Y: Mm. And continuity.” (2 A)*.

It was perceived as impossible to hurry planned actions or activities. The staff discussed that it would be more appropriate to conduct specific interventions when some time had passed, and PwD’s self-confidence had increased. Successively, there was a more positive attitude towards the intervention among the participants and then they saw it as an appropriate time to capture the least motivated and to motivate and plan for future activities. Additionally, they thought that time was needed for PwDs and their caregivers to achieve new insights and to notice improvements achieved. To have time to meet a PwD often, in her/his home, made an interplay possible, to increase involvement.

*“To take the time to do it … otherwise it might be something you have to ask others to do because you don’t have the time. And to get that interaction and apply yourself and your knowledge in how to guide her to feel that she is doing what she can do and feel involved. It was awesome!” (1B)*.

Since the same staff met the PwDs and their caregivers during the rehabilitation period, continuity could be achieved. The staff concluded that the intense contact, meeting in a small group in a safe environment, and being familiar with the staff members facilitated recall and created safety for the PwD. This was especially important for those anxious or sensitive to external disturbances, for example, that they had someone familiar who answered phone calls or welcomed and cared for them at the rehabilitation unit. The PwDs and the caregivers participating in the group meetings also got to know the other participants in their groups.

*“I think a lot of the time has been needed to create good relations and form a memory for these persons. So even those with short-term memory that you meet later… you come in and they say, ‘But hey, I recognize you’… and I don’t think that feeling would have existed if we had only met for a short period.” (3A)*.

### Creating a holistic view through knowledge exchange

The staff team expressed that they were *being strong together* through the stimulating teamwork. Their knowledge exchange was described as *to carry each other’s skills for the benefit of the clients* and that they were *getting an overall picture of participants’ needs*. Their *cooperation was refined over time* and thereby they were able *to achieve consensus and bringing a unified message* to the PwDs and their caregivers, which was perceived as important.

To work in a comprehensive team was expressed as fun, developing and timesaving. The staff team members learned about and supported each other and perceived this as giving energy and fellowship as well as power to the interventions. For some, it was a new experience to work in such a comprehensive team and so closely together, leading to a change in their process thinking and way of working.

*“X: Yes, absolutely. It catches people… so this is really person-centered care and what the person needs. And then all professional categories are included, and you can capture all needs and you can offer the right things…whatever is needed.*

*Y: Mm. Proper rehabilitation… Because, if there is a piece missing, then it will not be like that. Then it will just be different interventions.*

*Z: Yes, it will be…*.

*Y: But if you have this coordination and plan and work … towards goals that are clear to everyone who is…around this person, then it will be easier to get there as well.*

*X: There will be increased power in the interventions as they come from several sources.” (3B)*.

The staff team reasoned about how different professionals’ knowledge were exchanged in conventional meetings where the rehabilitation strategies were set up. Additionally, that there was knowledge exchange in unplanned meetings and chats, ‘*To carry each other’s skills’* were highly valued since this benefited both PwDs and informal caregivers. They believed it was especially important that more staff team members met a participant when the situation was more complex and problematic. Knowledge exchange was highlighted as necessary because the PwDs and their informal caregivers expressed different problems to different professions. One example of how to pass information to other professions was if one of the staff met a participant during a home visit and another during the exercise sessions, and they could act as *‘each other’s eyes and ears’.* Various and shared information and signals were important to *‘paint the whole picture’* and weigh all information together for the whole team.

*“You have the information in a completely different way, which makes it easier to get a grip on what the problem really is and where to try to solve it, so to speak. It is a completely different collection of information… You get more information if there are different professions than if you were to see the doctor five times in a row because it depends on who you meet, what profession it is, so you tell a slightly different thing.” (1 A)*.

The staff team adapted themselves and their input, for example based on the actual problems, shifting needs and daily form of the participants. They emphasized that the teamwork had to be flexible and customizable if the rehabilitation should be person-centred. This could mean that they gradually adjusted the interventions to succeed, for example, by adapting their planned activities or goals. It also included obtaining information from different contexts, e.g., during activities or exercise, during the social moment when having coffee together, or in the home environment. This was most evident for those living alone.

*“X: The environment provides incredible clues to how the person does at home and which activity level they have. If there is a lot of stuff on view, then you understand that they are still active. But if everything is removed, then you might think that … well, there might not happen that much here. You can interpret a lot based on what you see around you.*

*Y: If it is so well removed that you don’t see the things, you don’t get a reminder to do it…*.

*X: Exactly.*

*Y: Yes, everything is gone … You don’t see what you should do because it is not there.” (2 A)*.

The importance of the team conferences was emphasized, since these were the occasions where all staff team members met and could raise diverse perspectives of the participant’s situation and discuss common rehabilitation strategies. The staff team achieved increased understanding by illuminating problems from different points of view and *‘stitching the parts into a whole.’* This knowledge exchange was expressed in the focus groups as *‘Everyone’s knowledge in one pot,’ ‘Like adding puzzles,’ and ‘Like a palette.’* Through communication, the staff team gained expanded substance and insight into other professions’ work assignments, received confirmation of their own thoughts, and provided deeper understanding about the PwDs. By this working method they concluded that they got a clear overall picture of participants’ needs and that an interdisciplinary approach is required for holistic thinking.

“*If the patients’ problems are highlighted from different points of view and interventions are implemented from different angles and based on the goals…Yes, the same goal, we have the same goal that the patient should feel better. As an overall picture of the patient’s needs, I think.”(2B)*.

Characteristics of the teams that were highlighted as important were that there were no territories or hierarchical structure. Additionally, certain individual qualities among the staff team members that enabled and facilitated the teamwork were that they were experienced and generous, meaning, for example, that they were not prestigious when it came to sharing their expertise. The collaboration in the teams was refined over time when the staff team members learned to know each other and the study participants.

*“Just a little refined anyway…. I mean that maybe you got a little more energy and focus on the collaboration when the structure was ready. And as you can concentrate, it will probably get better.”(1B)*.

Also, collaboration and knowledge exchange were emphasized as invaluable to achieve a common perception. In turn, this was stated as important to convey a unified message to the participants in the rehabilitation programme, which was expressed as time saving, as well as being in the best interests of the participants and bringing them a feeling of safety.

*“That you have a team to confide in… because I mean, if you throw out a question in a team, there is always someone who can answer it, who says that ‘I know… you can do this. You can turn there.’ It’s also an incredible time saver.” (2 A)*.

## Discussion

The staff team’s experiences that are presented through the core category of the present study: ***Refining a co-creative process towards making a difference***, resemble the collaboration that the staff had within their teams during the rehabilitation period and towards their goal that the intervention should make a difference for the PwDs and their caregivers. The flexibility that the staff team shows runs like a red thread across the categories. To be able to collaborate, make adaptations, and find new solutions to challenges, the staff team members had to be open and act flexibly and have a flexible view on their own and others’ areas of expertise. Their flexibility is shown in their mediating role and in how they involved the participants, which was possible because of enough time and a collaborative and open-minded teamwork. The interpretation is that in our context the staff team, the PwDs and their informal caregivers formed the person-centred and individualised interventions in a co-creational process. According to Sanders & Stappers (2008) co-creation refers to any act of collective creativity. Under the name participatory design, collective creativity in design has been practiced for nearly 40 years. Today co-creation is also taking place among healthcare professionals in the design of new healthcare systems and environments [30]. Involving patients in service improvement and listening and responding to what they say has played a key part in the redesign of healthcare processes [31]. Sometimes patients and family members also become part of the design team. A design can focus on designing for a purpose, such as, design for experiencing, design for sustainability or design for transforming [30]. In our study a co-creative process is taking place within the teams (staff, PwD and caregiver) and a rehabilitation programme.

The teamwork involved active participation of both the PwDs and their caregivers and many different ‘realities’ to consider during the rehabilitation period, which required flexibility and creativity from the staff team. Their strategies differed depending on the situation, issue or constellation of persons or organizations the team would link to. This flexible approach required responsiveness and a positive attitude when working in a team and with older adults with dementia. The staff discussed the importance and the challenges that involvement of the PwDs in their rehabilitation constituted, which was most evident when it came to common goal setting. Like our findings, clinicians have previously found it challenging to involve patients, who have problems with communication and cognition, in goal setting [32]. According to WHO, the core of rehabilitation should be person-centred care that includes empowerment and goal setting [5]. In rehabilitation, goal setting is regarded as an essential aspect and has been defined as the establishment or negotiation of rehabilitation goals and refers to a change and the intended future state of the patient [33–35]. In a study where patient-centred goals in dementia care were elicited, participants with dementia articulated the need to readdress goals as the disease progressed. Henceforth, the authors concluded that patient-centred goals should be incorporated in clinical settings and that their usefulness for dementia care should be assessed [36]. Although findings about the effectiveness of goal setting in rehabilitation outcomes differ [37, 38], it has been concluded that using goal setting as an outcome is a sound measure for use in rehabilitation for older adults [39]. Within the framework of shared decision-making, goal setting may be considered desirable or even imperative from an ethical point of view, since goal setting involves patients in decision-making and is therefore a means to respect their preferences, values, and autonomy [38, 40, 41]. In Swedish laws it is stated that health care, as far as possible, has to be designed and implemented in consultation with the patient and the social services shall be built upon respect for people’s self-determination and integrity [42].

Clinicians in Australia also perceived barriers to participation in patients with dementia, according to an interview study [14]. Additionally, they acknowledged problems with existing dementia care pathways but rarely conceptualized rehabilitation as relevant to this pathway. They had difficulties defining worthwhile outcomes of a rehabilitation programme for people with dementia and believed that achievable outcomes were not sufficiently worthwhile for investment [14]. In contrast to these stereotypes, the staff in our study had a different attitude, showing that instead, they saw opportunities rather than obstacles. This might be affected by their mission of carrying out the rehabilitation in the best way. Active and engaged willingness among staff is, beside sufficient knowledge, of most importance to successfully implement new practices, according to the concept of Readiness for organizational change [43, 44]. The concept can be viewed as an individual psychological state, comprising lack of time and energy or fear of the consequences [44–47]. When seen as an organizational or collective characteristic there are several important focuses that can be recognized in our study, such as effective group decision-making processes [48], communication [49], programme coherence, and organizational climate [44].

The findings of the focus groups show that the staff valued having more time available for the participants than they usually have in their clinical work, and that time was a prerequisite for implementing and refining interdisciplinary teamwork. The teamwork was also described as time saving. In contrast, lack of time present within the health care system is described as creating stress of conscience, which is a certain kind of stress syndrome caused by bad conscience in relation to fall short of expectations and demands [50]. On the other hand, person-centeredness, support, and respect from clients and their families might have a counteracting effect on stress of conscience [51]. These characteristics were all present in the intervention of this study.

Interdisciplinary teamwork is recommended for patients with complex problems, as is the case for older adults with dementia [1]. According to Wade 2020, effective rehabilitation depends on a multidisciplinary expert team, working within the biopsychosocial model and working collaboratively toward agreed goals [35]. This is the approach in our rehabilitation programme. The focus groups emphasized the power of working in a team with access to many different professions, which justifies the need of well-staffed rehabilitation teams to meet the complex needs in this population. The staff enjoyed working together and described that they became stronger and more competent when collaborating. This implies sustainability in terms of sustainable staff, knowledge, and rehabilitation programmes. Although the staff had individual strengths, were well educated, or had long working experience they expressed that they needed the others in the team to continuously learn from each other and thereby get a holistic view. This is supported by the statement that there is a need for continuing training in areas outside each person’s limited professional field to acquire and maintain specialist expertise [35].

There are similarities to our findings in two studies about physiotherapists’ reflections of meeting older adults with dementia in the clinic [6] and in an exercise study context [52]. In these studies, emphasis was placed on the opportunity for reflection, skilled and adapted communication, the importance of knowledge and learning to build a trusting relationship, and on communication with the nursing staff [6, 52]. Additionally, the need for support and education for relatives was pointed out [6].

The staff team aspired to make a difference for each PwD and caregiver. In interviews with PwDs in the present rehabilitation programme, it was confirmed that the rehabilitation had made a difference. The informants described that they had been strengthened by the challenges posed by the rehabilitation and interaction with others, and that participation was worthwhile. As expressed in the overarching theme of the study, they were empowered through participation and togetherness [19]. If the team of staff members perceived the rehabilitation programme as effective, it might have strengthened their engagement and work satisfaction. The staff’s perceptions of the programme’s effectiveness must be studied further.

### Methodological considerations

The data collection was performed several times over a long period, which made processing of the data possible. There were no changes in the constellation of the focus groups, i.e., the participants were the same in every interview session, but the constant comparison resulted in changes in the interview guide from the first focus group interview to the second. Both an insider and an outsider perspective were represented within the research group that consisted of various professions with different knowledge-base and pre-understandings [23]. We performed parallel coding, triangulation between researchers, and comprehensive discussions in the whole research group, which likely contributed to a more nuanced discussion and data analysis, since the researchers have diverse backgrounds and experiences. Peer debriefing [23, 53] was achieved by presentations and discussions among colleagues and other health professionals who found the results credible. Therefore, we consider our findings to be trustworthy.

A limitation of the study might be that the agreement in the focus groups was evident and accordingly there was not much arguing or different opinions. Instead, the staff reaffirmed each other during the focus groups, which the quotes in the results section reflect. There might be a risk that the participants were too regardful and did not want to disagree. Additionally, the participants were all involved in offering the rehabilitation programme. This might mean that they were highly motivated and engaged in their work and wanted to achieve the best outcome for their clients. Their in-depth reflections and the rich interview data might be a result of their position.

### Implications for practice and future research

Knowledge about the team of staffs’ experiences of providing rehabilitation is essential to develop best practice. It may also positively impact attitudes and broader acceptance of rehabilitation as relevant for older adults with dementia. Important features to consider are collaborating within the team and with other actors, involving rehabilitation participants, creating relations, and having a flexible and generous approach. Further, the timing of goal setting, enough time, and forums for team discussions are vital.

Future research should focus on the effects and feasibility of a person-centred multidimensional inter-disciplinary team rehabilitation programme for community-dwelling older adults with dementia and their caregivers. The informal caregivers’ experiences of participating in the rehabilitation programme, and staff perceptions of what changes the programme entailed remain to be analyzed.

## Conclusions

According to staff team experiences a comprehensive team is viable to provide person-centred, multidimensional, and interdisciplinary rehabilitation for older adults with dementia and their informal caregivers. The team of staff could provide individualised rehabilitation in creative collaboration with the participants through interaction, knowledge exchange, time and continuity, coordination and flexibility, and a holistic view. Challenges to overcome were the involvement of the person with dementia in goal setting and the mediating role that the staff had to have. The staff pointed out that by refinement they could achieve well-functioning, competence-enhancing and timesaving teamwork.

### Electronic supplementary material

Below is the link to the electronic supplementary material.

Supplementary Material 1

## Data Availability

The datasets generated and analyzed during the current study are not publicly available to protect the participants’ confidentiality (in accordance with The General Data Protection Regulation, European Union Regulation) but are available from the corresponding author on reasonable request.

## Abbreviations

WHO: World Health Organization
ADL: Activities of daily living
GT: Grounded Theory
MIDRED: Multidimensional InterDisciplinary REhabilitaion in Dementia
PwD: Participants with Dementia
HIFE: High Intensity Functional Exercise
COREQ: The Consolidated Criteria for Reporting Qualitative Research
