# Supplementary material for: A focus groups study of staff team experiences of providing interdisciplinary rehabilitation for people with dementia and their caregivers—a co-creative journey
Source: BMC Geriatr. 2023 Sep 18;23:572. doi: 10.1186/s12877-023-04269-3 (PMC10507912; doi:10.1186/s12877-023-04269-3)
Supplement: Supplementary file 1 — Supplementary Material 1 [file 12877_2023_4269_MOESM1_ESM.pdf]

Supplementary file 1 – Interview guide

Topics in the semi-structured interview guide used with staff team providing rehabilitation to older adults with dementia, including education and support for informal caregivers

Older adult with dementia and primary caregiver

Tell about :

- a situation where the team work made a difference to the better
- when the team was especially important to the older adult
- when the team was especially important to the caregiver
- a situation when it was diffucult to make a difference

Describe the cooperation between the older adult – the team – the caregiver

Teamwork

Cooperation in the extended team
Working with many professions
Learning experiences:

- from other professions and over time
- own reflections on teamwork during the [illegible]

Experiences

Joy – Challenges - Frustrations
Take home experiences

Expectations on the teamwork project

Expectations on the project when starting
Did it turn out as you expected?
